# High-level biocidal products effectively eradicate pathogenic γ-proteobacteria biofilms from aquaculture facilities

**DOI:** 10.1016/j.aquaculture.2020.736004

**Published:** 2021-02-15

**Authors:** Félix Acosta, Daniel Montero, Marisol Izquierdo, Jorge Galindo-Villegas

**Affiliations:** a Grupo de Investigación en Acuicultura (GIA), IU-ECOAQUA, Universidad de Las Palmas de Gran Canaria, Crta. Taliarte s/n, Telde, Las Palmas, Canary Islands, 35214, Spain; b Faculty of Biosciences and Aquaculture, Nord University, Bodø 8049, Norway

**Keywords:** Aquaculture, Biofilms, Bacterial hazard, Disinfection, Gilthead seabream, γ-Proteobacteria, Peracetic acid, *Vibrionacea*

## Abstract

The use of effective biocides as disinfectants is essential in aquaculture facilities. However, while most biocides act effectively on free-living planktonic pathogens, they are seldom useful against biofilms. In this study, we evaluate the biocidal efficacy and antimicrobial specific contact time of three disinfectants, Virkon™Aquatic (VirA), peracetic acid (PerA) and hydrogen peroxide (HydP), on *Vibrio anguillarum*, *V. harveyi*, *V. alginolyticus*, and *Photobacterium damselae* subspecies *piscicida* against their both life phases. By using the minimum inhibitory, bactericidal, and eradication concentrations of disinfectants acting on the free-living planktonic state (MIC; MBC) and biofilms (MBIC; MBEC), we determined the *in vitro* susceptibility of each bacterial strain against three different individual concentrations of VirA, PerA, and HydP added at 1, 5, and 10 min intervals. PerA and VirA had the highest bactericidal efficacies against the free-living planktonic state and biofilm of all bacteria. Kinetically, PerA gave a positive result more quickly in both cases regardless of the strain in question, while the weakest HydP required longer than 10 min to act effectively. Moreover, we conducted a short *in vivo* safety trial by pouring the suggested MIC of each disinfectant into tanks containing juvenile Gilthead seabream (*Sparus aurata*). A significant mortality after 24 h was observed pointing to the potential risk a mishap of these chemicals might cause to fish. Nevertheless, collectively, our results support the inclusion of biocides within biosecurity protocols in aquaculture facilities and highlight PerA as the most effective disinfectant for fighting against biofilms produced by *V. anguillarum*, *V. harveyi*, *V. alginolyticus* or *P. damselae* subsp. *piscicida*.

## Highlights

• We explored the effects of three disinfectants against four pathogenic bacteria affecting most European warm water cultured fish.
• Virkon A (VirA) and hydrogen peroxide (HydP) have the lowest performance against biofilms.
• Peracetic acid (PerA) is the paramount disinfectant against both lifestyles of the four pathogens tested.
• Despite the thread that a disinfectant mishap may represent for live fish, their rational inclusion in biosecurity plans is recommended.

## 1 Introduction

Aquaculture is recognized globally as the fastest growing food-producing sector for human consumption (Bayliss et al., 2017). Notably, the European aquaculture sector alone provides over 3 million tons of fish annually (FAO, 2020). However, 90% of the European marine finfish aquaculture production comes from only seven countries (France, Greece, Italy, Norway, Spain, Turkey, and the United Kingdom). At the northernmost extreme, the salmon industry entirely dominates the scene, while in lower latitudes, large amounts of seabass (*Dicentrarchus labrax*), seabream (*Sparus aurata*), turbot (*Psetta maxima*), and, recently, meagre (*Argyrosomus regius*) are successfully produced (Miccoli et al., 2019). However, to satisfy increasing international demand, it is predicted that current production must be doubled by 2030 (Leduc et al., 2018). Such an expansion will necessarily involve implementing innovative actions in aquaculture production while also targeting effective health control measures and respecting animal welfare. Unfortunately, regardless of geographical location, species, or production level, diseases remain a significant bottleneck in fish farming.

Several marine and freshwater aquatic species inhabiting warm waters exhibit high sensitivity to the canonical pathological manifestation of vibriosis and photobacteriosis septicemia (Fatima et al., 2020; Galindo-Villegas et al., 2013; Remuzgo-Martínez et al., 2014). The genera *Vibrio* and *Photobacterium* are Gram-negative γ-proteobacteria included in the family *Vibrionacea* (Pečur Kazazić et al., 2019a; Vezzulli et al., 2013). In fish, the most critical pathogenic causative agents of vibriosis and photobacteriosis are three species of *Vibrio* (*V. anguillarum*, *V. harveyi*, *V. alginolyticus*), and one *Photobacterium* (*P. damselae* subsp. *piscicida*) (Igbinosa, 2016; Pečur Kazazić et al., 2019b). Although several studies exist on these four species, they are still regarded as an imminent threat to southern European fish aquaculture. Indeed, Vibrios are highly abundant in aquatic environments, including estuaries, marine coastal waters and sediments, and aquaculture settings worldwide are natural constituents of freshwater, estuarine, and marine environments (Thompson et al., 2009). Although these pathogens may be genomically diverse, they all originate from aquatic and marine environments. They prefer warm, salty water, and their abundance in the natural environment tends to mirror environmental temperatures (Baker-Austin et al., 2018). *Photobacterium damselae* subsp. *piscicida* is an intracellular fish pathogen that causes photobacteriosis, a disease proven deadly in farmed fish worldwide. Besides, *Vibrio* spp. and *P. damselae* subsp. *piscicida* are important fish pathogens presenting class 1 integrons and IncA/C plasmids that play a significant role in the transmission of antimicrobial determinants of antimicrobial resistance in the aquatic environment, which critically hamper fish culture activities (Miller and Harbottle, 2018). Concerning their living behaviors, they have variated lifestyles that could include a free-swimming planktonic state and a sessile existence that form biofilms attached to different surfaces, which enhances their survival and exponentially increases their infectivity capacity.

Two apparent reasons that play a central role in the transmission of the above-mentioned pathogenic bacteria to cultured fish are the farming activity, *per se*, and the open design of Mediterranean and Atlantic aquaculture systems. As such, the transport of potential pathogens from hatcheries, equipment, staff, visitors and vessels, and water currents and wild fish have been all seen as the main threat to fish health and associated biosecurity programs (Ina-Salwany et al., 2019). At present, fish are handled through a variety of biotic approaches involving immunostimulants, antibiotics, and vaccines, which are extensively used for the prevention, treatment, and long-term control of diseases respectively in aquaculture facilities (Galindo-Villegas et al., 2019; Montalban-Arques et al., 2015; Schmidt et al., 2017). However, in addition to these approaches, a rapid on-site cleaning and disinfection regime is a critical proactive strategy to control the spread of microorganisms colonizing fish farms.

Disinfection implies the use of biocides that provide antimicrobial activity and reduce the number of opportunistic bacteria, while some even have pathobiont capacities - usually associated with intimate matrixes (Meade and Garvey, 2018). Based on a range of disinfectant acting methods, these agents are extensively described elsewhere (Domínguez Henao et al., 2018b; Domínguez Henao et al., 2018a; SCENIHR, 2009; Tezel and Pavlostathis, 2015). Thus, all facilities should use biocides as disinfectants following best management practices (BMPs), which are normally detailed in dedicated biosecurity protocols on each farm. A wide choice of commercial biocides exists. Virkon™Aquatic (VirA), hydrogen peroxide (HydP), and peracetic acid (PerA) are three disinfectants with highly reactive sustainable properties that have been approved by the European Commission for health care and veterinary settings (Assefa and Abunna, 2018; SCENIHR, 2009). The variability that exists among the different formulas reflects their specific mode of action against microorganisms. For example, VirA oxidizes sulfur bonds in proteins and enzymes, altering the cell membrane's function and rupturing the cell wall. At the same time, the mechanism associated with the HydP and PerA relies on the powerful and direct oxidative disruption of cell membranes *via* hydroxyl radicals. Despite the widespread and successful use of these three disinfectants on the free-living planktonic stages of pathogens, little research has been done into their more complex bacterial associates, namely biofilms. Biofilms have a strong capacity to cause severe problems in aquaculture because they are a possible source of persistence, especially for multidrug-resistant bacterial lineages that have adapted to the facility settings (Günther et al., 2017).

The primary goal of our study was, therefore, to characterize the performance of VirA, HydP, and PerA against both the free-living planktonic state and biofilm formation of four pathogenic bacteria, namely *V. anguillarum*, *V. harveyi*, *V. alginolyticus*, and *P. damselae* subsp. *piscicida*. Following an *in vitro* approach, we demonstrate the effectiveness of the selected disinfectants, confirming that all three preparations can be recognized as plausible solutions for eradicating the free-living planktonic state of the pathogens in fish farms. However, and most importantly, we identify PerA as a practical solution against biofilm formation. A toxicity bioassay based on adding each disinfectant (at the suggested optimal concentrations as disinfectant) to tanks holding live juvenile Gilthead seabream (*Sparus aurata*) was carried out to determine the impact on the accidental fish spill of the same. Our work pointed to the clear advantages of applying disinfectants against the selected pathogens and supports the superiority of PerA for eradicating biofilms. However, caution applies to their use due to the trait the disinfectants represent after prolonged contact with the live warm water marine fish confined in intensive aquaculture facilities.

## 2 Material and methods

### 2.1 Bacterial strains and growth conditions

A panel of four pathogenic bacteria (*Vibrio anguillarum* L501, *V. harveyi* SA-1, *V. alginolyticus* SB-2, and *P. damselae* subsp. *piscicida* MED-1) were used in this study. The four strains were preserved at −80 °C in Brain Heart Infusion (BHI) medium (Pronadisa, Spain) mixed with 25% sterile glycerol as a cryopreserving element. Initially, every strain was aseptically cultured in sterile Erlenmeyer flasks containing 50 ml of BHI medium supplemented with 1% of sodium chloride (NaCl) following classical methods. Every container inoculated with a single colony-forming unit (CFU) of each bacterial strain was placed in an incubator (Selecta, Spain) at 25 °C for 24 h before use.

### 2.2 Disinfectant solutions

Three commercially available chemical agents that are regularly used as disinfectants were tested. A broad formula containing a mix of Pentapotassium bis(peroxymonosulfate) bis(sulfate), Sodium Dodecylbenzene Sulfonate, Butanedioic acid, 2-hydroxy-sulfamic acid, Potassium hydrogen sulfate, Sodium chloride, Dipotassium peroxodisulfate, and Dipotassium disulfate, namely, Virkon™Aquatic (VirA) was obtained from Bayer Laboratories, Spain (Cat.130000014173) and used at concentrations of 0.5, 1.0, 1.5%. Hydrogen peroxide 30% (HydP) was obtained from Sigma-Aldrich Chemical, USA (Cat. 216,763-M), and used at concentrations of 2.5, 5.0, 10.0%. The third solution consisting of peracetic acid (PerA) was obtained from Sigma-Aldrich Chemical, USA (Cat. 94,329), and diluted in seawater to working concentrations of 0.0005, 0.001, 0.005%.

### 2.3 Minimum inhibitory concentration assay

The minimum inhibitory concentration (MIC) of VirA, HydP, and PerA against *V. anguillarum*, *V. harveyi*, *V. alginolyticus*, and *Photobacterium damselae* subsp. *piscicida* was determined following the previously described broth microdilution method (Wiegand et al., 2008). Each disinfectant was diluted to the appropriate level with BHI supplemented with sodium chloride 1% (NaCl) Sigma-Aldrich Chemical, USA (BHIS). The microbial suspensions were diluted with BHIS to provide a density of 1 × 10^7^ CFU/ml. Then, 20 μl of diluted microbial suspension was added to the wells of polystyrene flat-bottom 96-well plate (Multiskan FC, Thermo Fisher) containing 180 μl of a disinfectant solution, thus providing a final concentration of 5 × 10^5^ CFU/ml. Three independent microplates for each disinfectant concentration were incubated for 24 h at 25 °C in aerobic conditions. In the final step, the resulting MIC of each disinfectant was spectrophotometrically determined at OD_600_ nm following classical protocols.

### 2.4 Determination of minimum bactericidal concentration

To determine the minimum bactericidal concentration (MBC), 50 μl aliquots from wells showing no visible growth in the MIC experiment were plated onto BHIS agar plates and incubated at 25 °C for 24 h. The MBC was measured as the lowest concentration of the chemical agents that killed 99.9% of the bacteria, and the level verified in cases where visible growth was absent (Zihadi et al., 2019). The final MIC and MBC values were defined from the same results obtained in three successive experimental sets.

### 2.5 Biofilm formation assay

The quantitative estimation of biolayer formation was performed by crystal violet (CV) staining, as described previously (Vivas et al., 2008). In brief, each bacterial strain's overnight culture was diluted to OD620 nm of ~0.05 in BHI, and 150 μl of these suspensions were pipetted into a U-bottom polystyrene 96 well plate and incubated under static conditions at 25 °C. During incubation, cells attached to the polystyrene formed a biofilm layer on the plates. After 48 h of incubation, the liquid medium above the biofilm layers was removed, and the wells were washed three times with generous amounts of sterile physiological saline. The resulting biofilm layers were stained for 12 min with 170 μl of CV (0.7%, *w*/*v*, solution) per well. Excess staining was removed by washing three times with distilled water. The plates were dried in a biological safety hood for 12 min, and then the CV was eliminated with 150 μl of 33% acetic acid. The plates were left 1 min at room temperature in an orbital plate shaker at 400 rpm to facilitate the removal of colorant. Next, 100 μl from each well was transferred to a flat bottom 96 microplate, and the amount of dye, proportional to the number of bacteria adhered, was quantified at OD_620_ nm in a plate reader (Multiskan FC, Thermo Fisher). Each value was subtracted from the control cell values, which only contained the culture medium. These experiments were carried out in triplicate, with eight wells per strain in each assay.

### 2.6 Determination of minimum biofilm inhibitory concentration (MBIC) and minimum biofilm eradication concentration (MBEC)

MBIC and MBEC experiments followed a previously reported method (Reiter et al., 2013). In brief, 20 μl of bacterial suspensions at a density of 1 × 10^8^ CFU/ml were added to 180 μl BHIB, placed into a flat bottom 96-well polystyrene microtiter plate, and incubated at 25 °C for 24 h without shaking to permit bacterial attachment. The supernatant was removed from each well, and the microplates were rinsed twice with 150 μl PBS. For each chemical agent, dilutions were prepared in BHIS using a fresh microtiter plate. To each well of the original plate containing the biofilm, fresh dilutions of VirA, HydP, and PerA were added. The plate was incubated at 25 °C for 24 h. After incubation, the lowest chemical agent concentration showing no growth after exposure to biofilm was recorded as MBIC. After measuring the MBIC, the broth was removed, and the wells were washed twice with PBS. Finally, the chemical agent was added, and the mixture was incubated at 25 °C for 24 h. MBEC was determined as the minimal concentration of chemical agent that failed to allow the bacteria to grow after being eradicated. The MBIC and MBEC final values were established by obtaining the same results in three alternative experimental sets.

### 2.7 Time-kill analysis of free-living planktonic state and biofilms

The killing activity of VirA, HydP, and PerA against the free-living *V. anguillarum*, *V. harveyi*, *V. alginolyticus*, and *Photobacterium damselae* subsp. *piscicida* was evaluated. For this purpose, bacterial suspensions diluted in sterile seawater at a final density of 1 × 10^8^ CFU/ml were prepared. In each procedure, 1.0 ml of the interfering organic matter (10 g/l yeast extract plus 10 g/l bovine serum albumin solution) was added to 1.0 ml of a bacterial test suspension in a sterile glass container. After adding 8.0 ml of each disinfectant at the previously determined MBC, the mixture was vortexed and incubated at 25 °C for 1, 5, and 10 min ± 3 s. The killing activity of the three disinfectants on the biofilms was assessed on 20 μl of bacterial suspensions at a density of 1 × 10^8^ CFU/ml added to 180 μl BHI media, placed in a flat-bottom 96-well polystyrene microtiter plate, and incubated at 25 °C for 24 h without shaking to allow bacterial attachment. The supernatant was removed, and the plates were rinsed twice with 150 μl PBS. In parallel, each disinfectant's dilutions were prepared in BHI media and added to the wells containing biofilm. In both cases, a colony count was made on BHI agar plates. The CFU/ml was transformed into log_10_ CFU. Control tests were processed following a similar protocol and excluding the use of the disinfectant product.

### 2.8 Ethics approval

All procedures with the fish complied with the guidelines of the European Union Council (86/609/EU) and Spanish legislation (RD 53/2013) and were approved by the Bioethical Committee of the University of Las Palmas de Gran Canaria (OEBA-ULPG-03/2020). Importantly, the number of animals used was determined following a highly restricted *f* size *a priori* effect established at the 0.05 α-error probability on the Power analysis accomplished with the GPower software (Meurs, 2016).

### 2.9 *In vivo* fish toxicity test

120 healthy juvenile (20 ± 1.0 g) Gilthead seabreams (*Sparus aurata*) obtained from a local facility were randomly distributed in 12 fiber-reinforced plastic (FRP) triplicate tanks of 50 l at the ULPGC facilities. Fish could acclimate for seven days before the start of the trial. We mimicked the accidental presence of VirA, HydP, PerA, as residues in tanks after disinfection by pouring 1.0, 5.0, 0.001, and 5 (%) of each disinfectant, respectively directly into the water of triplicate tanks. Additionally, 5% of sterile seawater was used as a mock control. Fish were monitored every two hours for data collection and to remove dead fish. The trial was stopped after 24 h, irrespective of the number of fish remaining in each tank.

### 2.10 Statistical analysis

Surviving populations of bacteria were reported as the median CFU/ml from three replicates. The CFU/ml obtained were transformed into log_10_ CFU before applying Tukey's Honestly Significant Difference (HSD) tests (Curran-Everett, 2018) to compare the effect of the different disinfectant types and exposure times on the reduction of bacterial growth. Survival data were evaluated by the Kaplan-Meier method. For all multiple comparisons, One-way ANOVA followed by Tukey's *post hoc* test was used. Statistical significance was accepted when *p* ≤ 0.05. Statistical analyses were performed using GraphPad Prism version 8.4.2 for macOS (GraphPad Software, San Diego, Cal, USA).

## 3 Results

### 3.1 Determination optimal dose of disinfectant

**Fig. 1:**
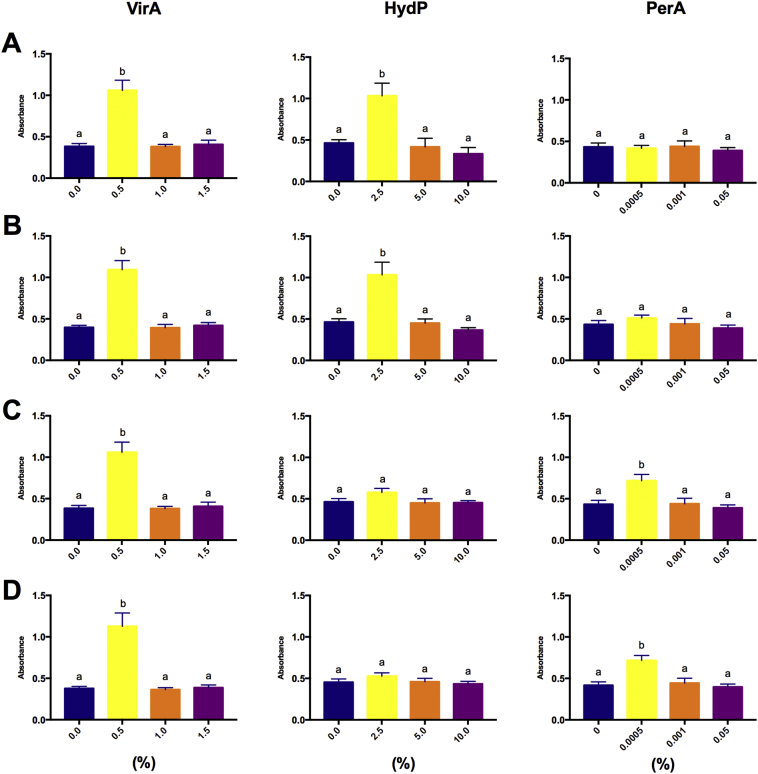
Dose-effect determination of selected disinfectants on each bacterial strain. Live bacterial suspensions at a final concentration of 5 × 10^5^ CFU/ml were exposed to graded concentrations (%) of disinfectants, from left to right: Virkon™Aquatic (VirA; 0, 0.5, 1.0, 1.5), hydrogen peroxide (HydP; 0, 2.5, 5.0, 10.0) and peracetic acid (PerA; 0, 0.0005, 0.001, 0.05). The pathogenic bacteria are represented by: (**A)***Vibrio anguillarum*, (**B)***Photobacterium damselae* subsp. *piscicida*, (**C)***Vibrio harveyi*, and (**D)***Vibrio alginolyticus*. Results are expressed as mean ± standard error of three replicates. Different letters represent significant (*p* ≤ 0.05) differences (One-way ANOVA with Tukey's *post hoc* test).

As a first approach in this study, the optimal dose providing (VirA, HydP, and PerA) with the ability to exert a practical antibacterial effect against the free-living planktonic states of *V. anguillarum* (Fig. 1A), *P. damselae* subsp. *piscicida* (Fig. 1B), *V. harveyi* (Fig. 1C), and *V. alginolyticus* (Fig. 1D) were assessed. Following the reference working concentrations, we chose three doses of each disinfectant to run the test. There was a significant (p ≤ 0.05) trend towards a reduction in cell counts when a concentration 2-fold higher than the lowest tested concentration. However, no change was recorded after that when the highest doses were compared. Validation was further accepted if each sample showed decreased absorbance within an acceptable absorbance range higher than 99.9% following triplicate observations for reproducibility. Nevertheless, the susceptibility of HydP for *V. harveyi*, and *V. alginolyticus*, and, on PerA for *V. anguillarum*, and *P. damselae* subsp. *piscicida* did not vary between the three concentrations. Although, irrespective of the latter observation, we determined that 1.0% VirA, 5.0% HydP, and 0.001% PerA were enough to inhibit the growth of free-living planktonic cells for all the pathogenic bacterial strains studied.

### 3.2 Minimum inhibitory and bactericidal concentrations of disinfectants against free-living bacteria

**Fig. 2:**
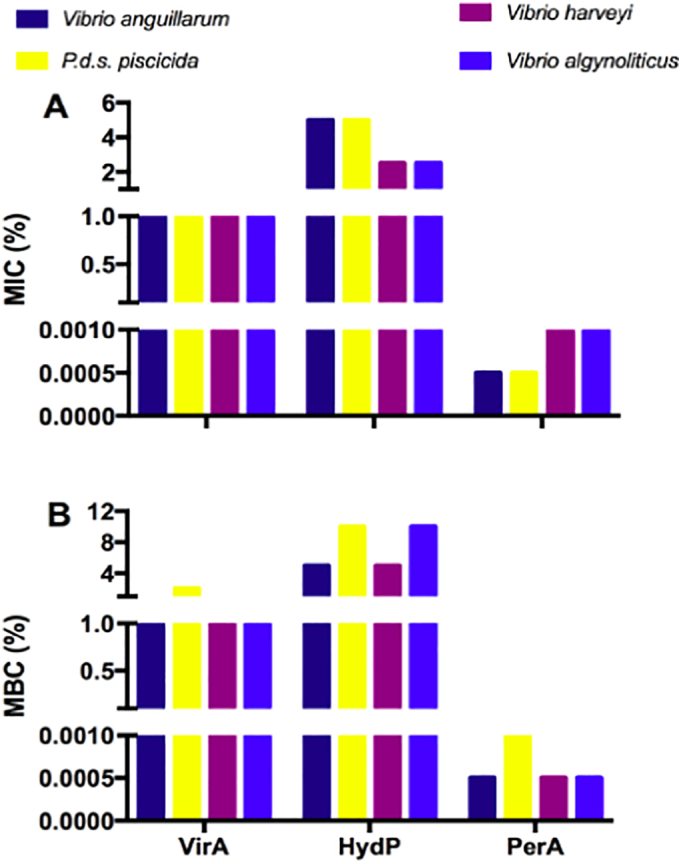
Susceptibility of the free-living planktonic state of the bacterial strains against disinfectants. (**A)** Minimum inhibitory concentration (MIC), and (**B)** Minimum bacterial concentration (MBC) of three commercial disinfectants, Virkon™Aquatic (VirA; 1.0%), hydrogen peroxide (HydP; 5.0%) and peracetic acid (PerA; 0.001%), against *Vibrio anguillarum*, *V. harveyi*, *V. alginolyticus*, and *Photobacterium damselae* subsp. *piscicida* bacterial strains. The results, expressed as (%), represent the lowest concentration showing no visible growth. Both assays, MIC, and MBC, revealed PerA as the best performing disinfectant against the four pathogens tested. Bars represent the mean values of three replicate experiments.

To determine the MIC values of VirA, HydP, and PerA, we applied the double-dilution method, based on determining the turbidity produced by increasing the bacterial density. We used the same bacterial targets and above described optimal doses for each disinfectant. Fig. 2 (A) shows no difference in susceptibility for VirA, which maintained a constant estimated MIC of 1% despite the bacterial strain tested. However, for HydP and PerA, we found an unusual antagonistic behavior. *V. anguillarum* and *P. damselae* subsp. *piscicida* had the highest estimated MIC value of 5% when subjected to the action of HydP, while *V. harveyi*, and *V. alginolyticus* displayed an estimated MIC of only 2.5%. By contrast, when *V. anguillarum* and *P. damselae* subsp. *piscicida* were subjected to the action of PerA, they showed the lowest estimated MIC value of 0.0005%, while the estimated MIC of *V. harveyi* and *V. alginolyticus* was double that (0.001%). These results suggest that PerA is the most potent disinfectant, with the highest susceptibility generation capacity and the lowest estimated MIC value of the three disinfectants tested against the four pathogenic bacterial strains under screening.

Next, we determined the MBC of VirA, HydP, and PerA, which effectively prevents the growth of *V. anguillarum*, *P. damselae* subsp. *piscicida*, *V. harveyi*, and *V. alginolyticus*. A colony count was made by pouring onto solid agar plates, the liquid culture used previously to estimate the least MIC for each disinfectant. Fig. 2 (B) shows how PerA was the best performing and most effective disinfectant. The MBC achieved by PerA was 0.0005% and 0.001% for the three *Vibrios* and the *Photobacterium*, respectively. In contrast to the low MBC shown by PerA, the VirA MBC baseline was reached at 1% against any of the three *Vibrios* tested. However, *Photobacterium* overcame the baseline level and displayed an MBC of up 4%. At the same time, the action of HydP against *V. anguillarum V. harveyi* had an MBC of 5%. In comparison, *Photobacterium* and *V. alginolyticus* showed the highest MBC by increasing the threshold to 8%. Overall, PerA positioned itself as the most effective disinfectant since it required the lowest inhibitory dose and highest bactericidal effect against the four pathogens tested on their free-living planktonic state.

### 3.3 Bactericidal effect of MBCs of disinfectants in cultures after different exposure times

**Fig. 3:**
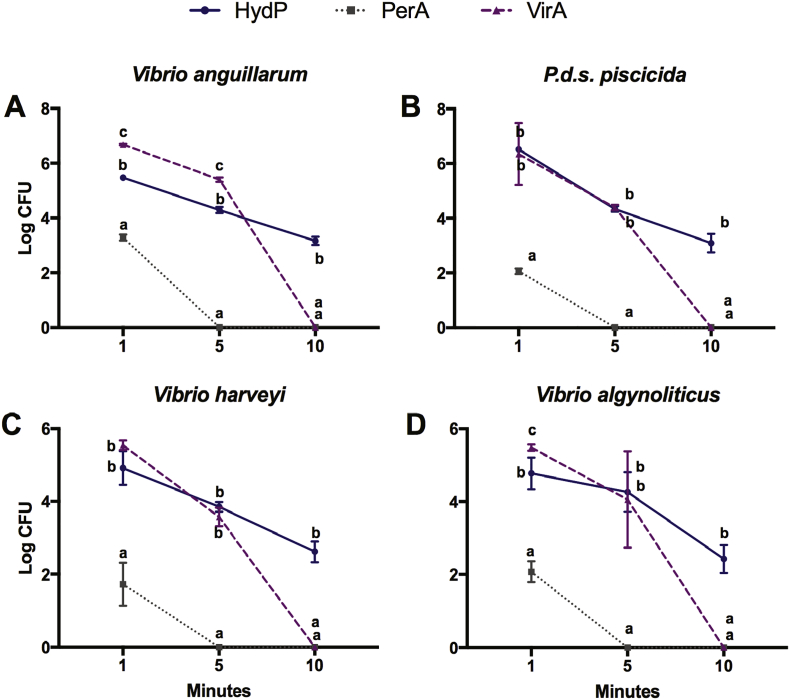
Free-living bacterial eradication kinetics shown by disinfectants against the four bacterial strains. Graph shows the eradication kinetics and the mean Log_10_ free-living viable counts (Log10 CFU/ml) of (**A)***V. anguillarum*, (**B)***P. damselae* subsp. *piscicida*, (**C)***V. harveyi*, and (**D)***V. alginolyticus* during 10 min exposure to Virkon™Aquatic (VirA; 1.0%), hydrogen peroxide (HydP; 5.0%) and peracetic acid (PerA; 0.001%) in three replicates. The three disinfectants were administered at the MBIC values previously determined. PerA performed better and faster than VirA and HydP. Different letters show statistical differences (p ≤ 0.05). n.s. (Not significant).

To further characterize the kinetic activity of the disinfectants, we studied the susceptibility effects at the free-living planktonic state of *V. anguillarum*, *P. damselae* subsp. *piscicida*, *V. harveyi*, and *V. alginolyticus* on liquid growth cultures following a time-course profile with intervals at 1, 5, and 10 min. The test was run using the previously determined MBC for each disinfectant and bacterial strain. As observed in **(**Fig. 3**)**, in the presence of PerA, the four bacterial strains significantly (p ≤ 0.05) reduced their reproduction rate after only 1 min. Furthermore, their growth was completely inhibited by the action of PerA after 5 min' treatment. The effect of VirA and HydP always was lower than that of PerA, inducing a similar rate of bacterial killing and growth suppression on the four bacterial strains after 1 or 5 min of treatment. However, by 10 min, VirA had entirely eradicated the four bacterial strains, while HydP was still at a lower level of a significantly (p ≤ 0.05) different inhibitory stage.

### 3.4 Biofilm formation ability

**Fig. 4:**
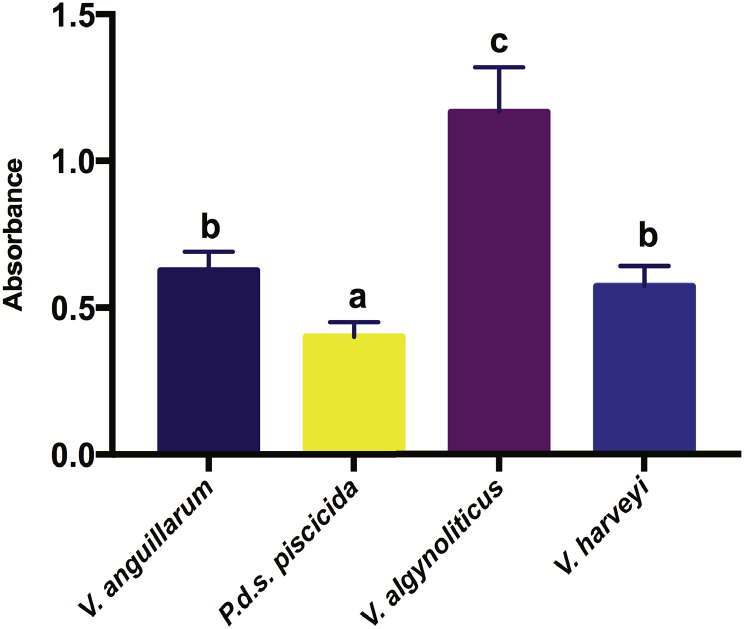
Biofilm-formation ability of each bacterial pathogen. Biofilm production analysis through crystal violet staining is expressed as the absorbance emitted by each of the four bacterial strains: *Vibrio anguillarum*, *Photobacterium damselae* subsp. *piscicida*, *Vibrio harveyi*, and *Vibrio alginolyticus* incubated in brain heart infusion (BHI). The absorbance of the observed values varied between (0.44) for *Photobacterium damselae* subsp. *piscicida* to (1.23) for *Vibrio alginolyticus,* while *Vibrio anguillarum and Vibrio harveyi* formed biofilms at intermediate values. Data represent mean ± SD for three independent experiments. (*n* = 3). Different letters denote the statistical significance between groups (p ≤ 0.05). (For interpretation of the references to colour in this figure legend, the reader is referred to the web version of this article.)

Next, to test whether the disinfectants effectively eradicate the complex bacterial associations, we proceeded to grow and characterize their biofilm-forming ability. Again, *V. anguillarum*, *P. damselae* subsp. *piscicida*, *V. harveyi*, and *V. alginolyticus* were cultured and submitted to crystal violet (CV) staining, successfully confirming the four pathogenic bacterial isolates as biofilm formers. However, according to the CV staining results, *V. alginolyticus*, *V. anguillarum*, and *V. harveyi*, and *P. damselae* subsp. *piscicida* presented significant (p ≤ 0.05) differences, and so we considered their biofilm formation ability as robust, moderate, and weak, respectively (Fig. 4). The number of live adherent cells forming the biofilm layer reached a significant peak in the robust *V. alginolyticus*, which displayed an absorbance value of 1.23, while the weak *Photobacterium damselae* subsp. *piscicida* had the lowest value (0.44 Abs).

### 3.5 Susceptibility of bacterial isolates in biofilms to the disinfectants

**Fig. 5:**
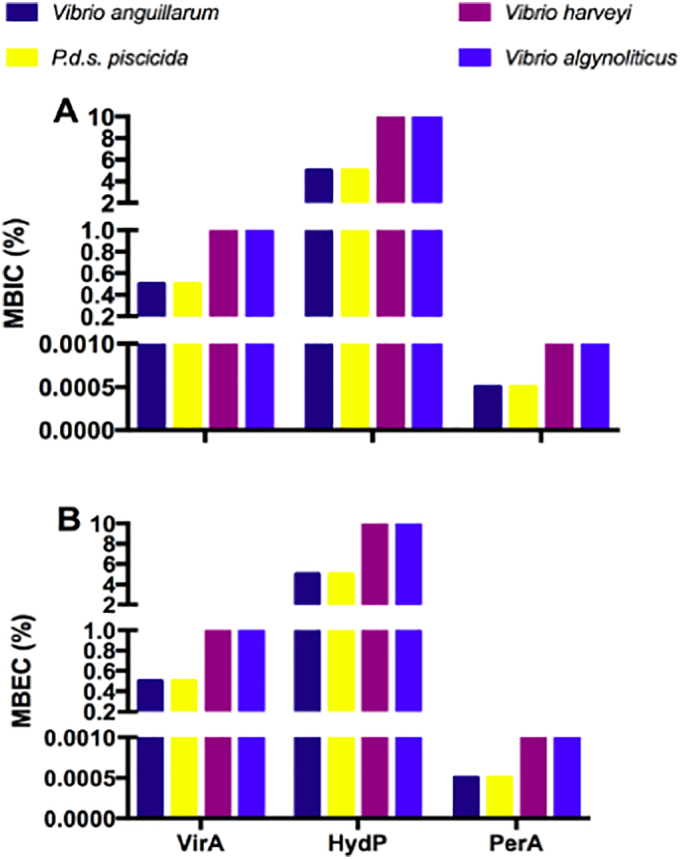
Susceptibility of the four bacterial strains to disinfectants. **(A)** Minimum biofilm inhibitory concentration (MBIC), and (**B)** Minimum biofilm eradication concentration (MBEC) of three commercial disinfectants Virkon™Aquatic (VirA; 1.0%), hydrogen peroxide (HydP; 5.0%) and peracetic acid (PerA; 0.001%) against *Vibrio anguillarum*, *V. harveyi*, *V. alginolyticus*, and *Photobacterium damselae* subsp. *piscicida* bacterial strains. The results represent the lowest concentration leading to no visible growth expressed as (%). Both assays, MBIC, and MBEC, revealed PerA as the best performing disinfectant against the four pathogens tested. The clearing of turbidity was determined visually. Bars represent the mean values of three replicates.

Using the above-optimized values, we assessed the minimal biofilm inhibitory concentration (MBIC) and minimum biofilm eradication concentration (MBEC) of VirA, HydP, and PerA against *V. alginolyticus*, *V. anguillarum*, and *V. harveyi*, and *P. damselae* subsp. *piscicida*. When subjected to the action of PerA, the four bacterial strains provided the same MBIC values as their respective MIC values. *V. harveyi*, and *V. alginolyticus*, behaved similarly, while *V. anguillarum* and *P. damselae* subsp. *piscicida* showed a 2-fold lower VirA MBIC than its MIC. Lastly, *V. harveyi*, and *V. alginolyticus* showed the most pronounced shift with a 4-fold increase, and *V. anguillarum* and *P. damselae* subsp. *piscicida* showed a modest 2-fold lower MBIC for HydP than its MIC (Fig. 5 A). Next, we explored the MBEC of the disinfectants. Biofilm eradication at the MBEC should be complete, meaning that no viable bacteria would survive to multiply and restore colonization. For the three disinfectants, we observed the same pattern. *V. harveyi* and *V. alginolyticus* had a higher MBEC than *V. anguillarum* and *P. damselae* subsp. *piscicida*. However, numerically, the differences observed among them were 10,000-fold between PerA and HydP, and 1000-fold compared with VirA (Fig. 5B).

### 3.6 Bactericidal effect of MBCs of disinfectants in biofilms after different times of exposure

**Fig. 6:**
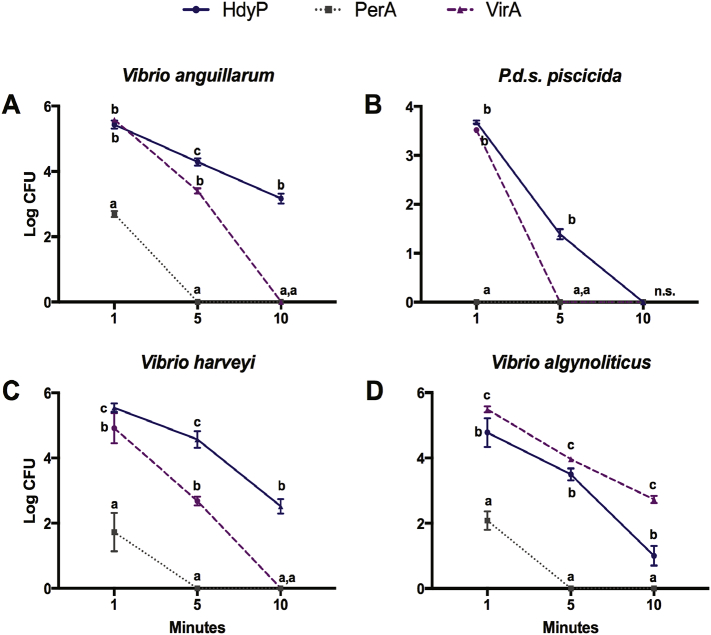
Biofilm eradication kinetics of disinfectants acting on the four bacterial strains. Graphic showing the biofilm eradication kinetics and the mean Log_10_ biofilm viable counts (Log10 CFU/ml) of (**A)***V. anguillarum*, (**B)***P. damselae* subsp. *piscicida*, (**C)***V. harveyi*, and (**D)***V. alginolyticus* during 10 min exposure to Virkon™Aquatic (VirA; 1.0%), hydrogen peroxide (HydP; 5.0%), and peracetic acid (PerA; 0.001%) in three replicates. The three disinfectants were administered at the MBIC values previously determined. PerA performed better and faster than VirA and HydP. Different letters show statistical differences (p ≤ 0.05). n.s. (Not significant).

We completed the series by further characterizing the disinfectant kinetic activity on the biofilm susceptibility at 1, 5, and 10 min. The amounts of the VirA, HydP, and PerA disinfectants used were the MBEC previously established for each group: 1.0, 10.0, and 0.001 (%), respectively. As observed in **(**Fig. 6**)**, the four bacterial strains significantly (p ≤ 0.05) reduced their reproduction rate after only 1 min in the presence of PerA, while the biofilm formed by *P. damselae* subsp. *piscicida* was so susceptible to contact with PerA that not even 1 min was required to eliminate it. VirA was the second-best performing disinfectant as seen from the significant (p ≤ 0.05) differences it showed in its action against HydP, especially after 5 and 10 min but only in the case of three bacterial strains, *V. anguillarum*, *P. damselae* subsp. *piscicida*, and *V. harveyi*. Surprisingly, the efficacy of HydP against *V. alginolyticus* was significantly (p ≤ 0.05) more potent than that observed for VirA throughout the 10-min time-course.

### 3.7 Toxicity of disinfectants *in vivo*

**Fig. 7:**
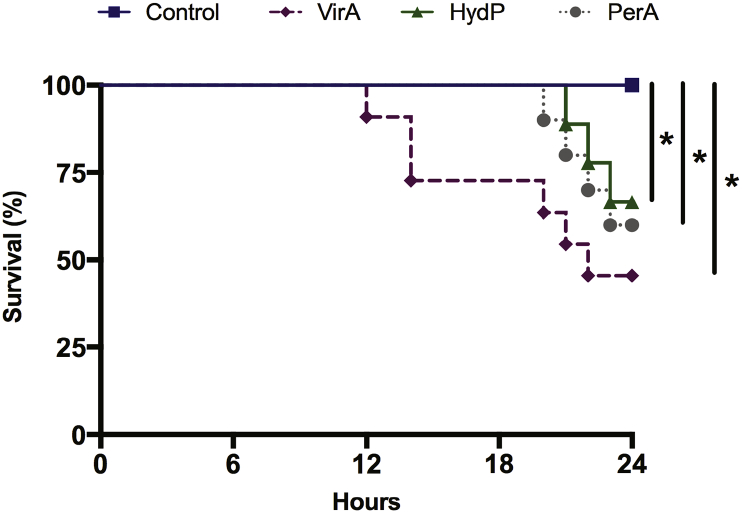
*In vivo* screen toxicity test using disinfectants*.* Juvenile gilthead seabreams were exposed to Virkon™Aquatic (VirA; 1.0%), hydrogen peroxide (HydP; 5.0%), and peracetic acid (PerA; 0.001%) poured directly into the water on the rearing tank. Mortality was recorded every two hours for one day. The concentration of each disinfectant is based on the MIC previously determined in this study. in addition, another group received 5.0% of autoclaved seawater as control. The three groups showed significantly higher mortality than the control group. Data are representative of at least three separate experiments (*n* = 10 animals per group). Survival curve evaluated by the Kaplan-Meier method. *p ≤ 0.05.

Finally, we exposed juvenile Gilthead seabream (*S. aurata*) to VirA, HydP, and PerA at the previously determined MIC. A significant (p ≤ 0.05) increase in mortality was observed in all the groups compared to the control after 24 h (Fig. 7). No statistical differences were recorded between the groups treated with disinfectants. However, the highest mortality rate observed (higher than 50%) was in the VirA group. Indeed, the fish subjected to the action of VirA start to die after only 12 h of exposure, while the fish treated with PerA and HydP did it so after 19 h of continuous exposure to the disinfectant at the doses applied.

## 4 Discussion

Biocidal disinfectant agents have attracted widespread attention due to their diverse active and potent mechanisms that directly disrupt pathogen cellular structure. However, until now, most disinfectants have been primarily studied for their effect on the free-living planktonic stage of bacteria (Günther et al., 2017). Although the collective sessile form of the bacterial growth generates a slime matrix of interdependent, persistent, attached bacteria forming a structured community capable of coordinating collective behavior (called a biofilm) requires alternative disinfection protocols.

In this study, we report on the successful use of disinfectants on the eradication of four free-living and biofilm-forming pathogenic bacteria (*V. anguillarum*, *V. harveyi*, *V. alginolyticus*, and *P. damselae* subsp. *piscicida*) that can cause disease in several farmed fish species, or which may even be fatal in immunocompromised specimens (Rowe et al., 2014; Sarropoulou et al., 2012)**.** Initially, our work provided insight into the optimal dose range requirements of three commonly used high-level biocides (HydP, VirA, and PerA) to inhibit the growth of the four bacterial strains in their free-living planktonic stages. We found marked differences in the amount of disinfectant required to eliminate each targeting pathogen. As in a previous work (Hawley et al., 2018; Liu et al., 2016), we observed that HydP requires a much higher inhibitory concentration when used alone than in the mixed formulas containing PerA, despite both biocides belonging to the same type of proxygene oxidizing disinfectants that function in much the same way. Indeed, the strong reactivity of PerA and HydP as disinfectants is known, and both these biocides have been seen to act as successful controllers of several pathogens recurrently found in fish farms in their free-living stages, such as *Piscirickettsia salmonis* (Muniesa et al., 2019), *Aeromonas spp*. (Craveiro et al., 2015), *Yersinia ruckeri* (Yamasaki et al., 2017), or even the noxious phytoplanktonic cells sometimes present in aquacultural recirculation systems (Liu et al., 2016). Interestingly, the action range of these biocides is broad and extends far beyond the marine environment. The inhibitory concentrations suggested here for PerA (0.001%) and HydP (5.0%) have been reported as being effective against *Vibrio vulnificus*, and *Salmonella typhimurium*, two deadly bacteria affecting a wide range of vertebrates, including humans (Salive et al., 2020).

In our work, by screening the inhibitory power of VirA, a broad-spectrum compound containing the strong surfactant SDS that acts by dissolving the plasma membrane of cells, we identified that it was effective at 1% dilution at inhibiting bacterial growth. Previous researchers using VirA also found that the same dose was capable of killing several free-living marine pathogens under high organic load conditions, such as those of fish farms environments (Love and Norley, 2020; Tidbury et al., 2018). Likewise, VirA was also recently reported to be effective in inhibiting the Saprolegnia's spore germination that affects common carp (Rahman and Choi, 2018). The all-round strength observed for our target disinfectants is not without importance. It goes a long way towards fulfilling the expectations held for biocides for eliminating contamination caused by a wide variety of pathogenic microorganisms in many different environments, as well as the associated organic matter that may harbor such organisms following repopulation. Whatever the case, VirA, HydP, and PerA, provide clear and convincing experimental evidence of possessing a low and multiuse MIC, two of the main features desired for any effective biocide (Maillard, 2018). Furthermore, the results showed a relatively stable MBC value among groups. However, on single bacteria, the MBC revealed specific features for each disinfectant-bacterial strain combination, while highlighting the variability in the response. The mean MBC value produced by *P. damselae* subsp. *piscicida*, with each disinfectant, suggests a degree of acquired resistance of the same or an enhanced killing function. However, further investigations may throw light on this disparity. Nevertheless, variability among different strains or even biotypes seems to be shared among these four pathogenic bacteria. Previous studies using VirA (Love and Norley, 2020; Rahman and Choi, 2018), or a variety of hydrogen peroxide-based disinfectants on a broad panel of Gram-positive and Gram-negative bacteria comprising *V. parahaemolyticus*, *Escherichia coli*, *Staphylococcus aureus*, *Pseudomonas aeruginosa*, and even *Saprolegnia* have reported reduced inhibitory profiles. However, variations among the bactericidal concentrations were recorded (Ríos-Castillo et al., 2017; Wong et al., 2018).

To gain a comprehensive understanding of each disinfectant's minimum contact time, we conducted a kinetic assay, including each bacterial strain. Once more, PerA was the first that significantly inhibited the viable cell numbers of *V. anguillarum*, *V. harveyi*, *V. alginolyticus*, and *P. damselae* subsp. *piscicida* that were present in each tube after just 1 min. Nevertheless, PerA eliminated all visible bacteria independently of the strain after a 5-min interval. Our data are broadly consistent with those of previous works using PerA on different models (Bounoure et al., 2006; Flores et al., 2016). Conversely, in our study, HydP and VirA took a significantly slower contact time response to achieve an effective full inhibition. Consequently, these results highlight that when choosing the most suitable disinfectant, the time necessary to neutralize pathogenic organisms affecting fish farms should be carefully scrutinized.

Unlike in the case of the free-living planktonic stages, our knowledge of the interactions between disinfectants and biofilms affecting aquaculture facilities is scarce. It is restricted to the knowledge that most biofilms are encased by extracellular polymeric substances that seem to be responsible for providing cells with widespread resistance to biocides (Cámara-Almirón et al., 2020; Díaz-Pascual et al., 2019). Indeed, very few studies have been specifically conducted on the resistance of *V. anguillarum*, *V. harveyi*, *V. alginolyticus*, and *P. damselae* subsp. *piscicida* biofilms to disinfectants (Elexson et al., 2014; Remuzgo-Martínez et al., 2014). For instance, we were able to visualize the biofilm formation ability of the four strains in our possession by monitoring the information obtained from the CV assay. Based on the three different biofilm strengths they produce; a gradient response was established. The pathological success of most bacteria lies in its virulence attributes. Interestingly, previous studies using *Enterococcus faecalis* and *P. damselae* subsp. *piscicida*, representing Gram-positive and Gram-negative pathogenic organisms, showed that differences in virulent metabolic activities are a significant determinant of the biofilm ability (Khouadja et al., 2014; Suriyanarayanan et al., 2018). The robust biofilm formation of *E. faecalis*, for example, arises from the significant up-regulation of the shikimate kinase pathway and sulfate transport. However, in the case of *P. damselae* subsp. *piscicida*, the hemolytic activity, and the adhesion profile marked the difference in the strength of the biofilms formed. Coincidently, on *V. anguillarum*, the modulation of the shikimate pathway through 3-deoxy-7-phosphohepatulonate synthase has been demonstrated (Guanhua et al., 2018). However, *V. anguillarum* is also dependent on iron and temperature for conditioning biofilm formation (Lages et al., 2019).

Our study focuses on the disinfectants required for the analysis of just one strain per bacterium. However, further investigations using various strains are warranted to elucidate the multiple abilities they have to form multispecies biofilms and, thus, the resulting virulence and susceptibility to disinfectants. Whatever the case, we found differences in the biofilm formation capacities among the four bacteria. These results demonstrate that our cellular matrixes were suitable for pursuing this trial's objectives, as described below. In the current study, the minimum concentrations required for the effective inhibition and eradication of the biofilms generated by *V. harveyi* and *V. alginolyticus* reflected the increasing resistance to PerA, VirA, and HydP than *V. anguillarum*, and *P. damselae* subsp. *piscicida*. Both analyses, the MBIC and the MBEC, revealed new information. Only the MBIC recorded for PerA was like the previously measured MIC, while for the rest of the disinfectants and strains, different response patterns were recorded. Nevertheless, of interest is the strength of PerA and HydP. In the treatment to determine the MBEC, PerA required 10,000 fewer units than HydP to achieve an effective eradication response in each of the four bacterial strains tested. However, the equilibrium achieved by PerA seemed to depend on the hydrogen peroxide present in the liquid formula (Flores et al., 2016). For VirA a similar response to that of the other two disinfectants in the MBIC and MBEC was observed. Nevertheless, VirA contains various chemicals that act synergistically with a myriad of mechanisms, making comparisons with previous studies difficult. The *Vibrio* spp. and *Photobacterium* sp. associated with surfaces and growing within biofilms are undeniably more resistant to biocides than planktonic cells (Barroso et al., 2019; Nonaka et al., 2015). Several virulence determinants of the *Vibrionaceae*, like quorum sensing, the production of lytic enzymes, or, more importantly, the ability to cohere to form biofilms are evolutive characteristics that must be overcome to achieve successful eradication (Austin and Zhang, 2006; Bunpa et al., 2020; Naka and Crosa, 2011; Terceti et al., 2019). Therefore, the use of biocidal disinfectants against bacterial biofilms and their eradication is not straightforward and critically depends on the wet time of contact.

To eradicate biofilms based on the MBEC achieved by the three disinfectants, HydP and VirA showed slower responses after 1 and 5 min. In contrast, the PerA significantly reduced the overall biofilm mass after only one minute and entirely deactivated the viable cells forming the *V. anguillarum*, *V. harveyi*, *V. alginolyticus*, and *P. damselae* subsp. *piscicida* biofilms after 5 min. Similar results demonstrating that PerA hindered adhesion and effectively controlled the biofilm formation of *Staphylococcus aureus* and *Salmonella* spp. were obtained previously (Iñiguez-Moreno et al., 2018). However, the time taken for biofilms to form is crucial, and it must be remembered that our biofilms were grown for only 48 h. Some studies have demonstrated significant variability in the amount of this disinfectant required to eradicate 24, 48, 96, 144, or 192 h-old biofilms in bacterial species such as *Listeria monocytogenes*, *Salmonella enterica*, or *Pseudomonas aeruginosa* (Akinbobola et al., 2017; Kostaki et al., 2012). Whatever the case, in addition to the chemicals included in each preparation and the biofilm age, several highly variable intrinsic factors like temperature, intra-species competition, or nutrient bio-ability actively contribute to the regulation of pathogenic bacterial outbreaks in aquaculture (Matanza and Osorio, 2018), affecting the effectiveness of biocides against biofilms (Cai and Arias, 2017). Therefore, it is necessary to find reliable strategies for each species and environment, like that successfully applied here based on the use of PerA to eliminate biofilms while minimizing the associated biosecurity risk they pose to the fish.

Finally, to support the application of the new prophylactic measures proposed for the high-level biocidal disinfectants in fish farms, a short trial with live fish was conducted, using as model animal the most widely cultured species in the Mediterranean region, the European Gilthead seabream. Although accidental contact with the biocides is highly unlikely, the fish were subjected to the action of VirA, HydP, and PerA at the concentrations suggested for a 24 h period. The results showed that the impact of disinfectants on fish health must be determined before they are used and that the concentration suggested for cleaning be strictly respected, even though the fish began to die after 12 h with VirA, and much later when the organic peroxides were used. In agreement with our findings, previous results with salmonids emphasized the need for care when using PerA (Gesto et al., 2018; Soleng et al., 2019) or HydP (Bowers et al., 2002) as routine disinfectants of free-living pathogens in recirculating aquaculture systems. Recently, Soleng et al. (2019) provided an immunological profile of Atlantic salmon smolts subjected to the action of PerA and described the mucosal and systemic stress responses they manifested as immediate defense mechanisms (Soleng et al., 2019). In contrast, Rahman and Choi (2018) tested the direct effect of VirA on the disinfection of common carp and reported no evident harmful effect (Rahman and Choi, 2018). Nevertheless, the authors suggest that caution should be exercised due to its possible bioaccumulation in treated animals. Whatever the case, our results demonstrate that to preserve fish health and welfare, contact with disinfectant agents should be avoided, and strict rinsing protocols should be followed each time they are applied. Moreover, aggressive contingency plans must exist and be acted on in case of any mishap involving disinfectants.

## 5 Conclusions

We have demonstrated marked differences among the three high-level biocidal disinfectants tested, although they are all, to a greater or lesser extent, capable of reducing the susceptibility of fish to pathogenic *Vibrionaceae* species regularly present in fish culture facilities. Overall, VirA and HydP showed lower efficacy, while PerA demonstrated a substantial added value by effectively fighting *V. anguillarum*, *V. harveyi*, *V. alginolyticus*, and *P. damselae* subsp. *piscicida* in their free-living planktonic stage. Nevertheless, it is worth noting that we discovered that a short 5-min exposure to 0.001% PerA effectively eradicates the more complex and resistant community association formed by each of these bacterial species, the biofilm. As discussed above, the seabream treated with the oxidizer PerA observed enhanced survival. We hypothesize that the enhancement was produced by mobilizing the systemic and mucosal antioxidizing defenses as reported previously in other species. However, this hypothesis requires of further analysis. Taken together, our findings provide support to the idea of including disinfectants in a biosecurity plan, despite their chemical origin. Lastly, the novel findings obtained for PerA highlight its potential role as biocide for all stages of the pathogens tested, from the onset of bacterial colonization to the more established biofilm found in fish farms.

## Declaration of Competing Interest

The authors declare that they have no known competing financial interests or personal relationships that could have appeared to influence the work reported in this paper.
